# Reelin Immunoreactivity in the Adult Spinal Cord: A Comparative Study in Rodents, Carnivores, and Non-human Primates

**DOI:** 10.3389/fnana.2019.00102

**Published:** 2020-01-08

**Authors:** Agnieszka Krzyzanowska, Marina Cabrerizo, Francisco Clascá, Tania Ramos-Moreno

**Affiliations:** ^1^Department of Anatomy and Neuroscience, School of Medicine, Autonoma University, Madrid, Spain; ^2^Division of Urological Cancers, Faculty of Medicine, Lund University, Lund, Sweden; ^3^Instituto de Investigación i+12, Hospital Universitario 12 de Octubre, Universidad Complutense de Madrid, Madrid, Spain; ^4^Lund Stem Cell Center, Division of Neurosurgery, Department of Clinical Sciences, Faculty of Medicine, Lund University, Lund, Sweden

**Keywords:** primates, spinal cord, motor neurons, nociception, reelin

## Abstract

Reelin is a large extracellular matrix (ECM) glycoprotein secreted by several neuronal populations in a specific manner in both the developing and the adult central nervous system. The extent of Reelin protein distribution and its functional role in the adult neocortex is well documented in different mammal models. However, its role in the adult spinal cord has not been well characterized and its distribution in the rodent spinal cord is fragmentary and has not been investigated in carnivores or primates as of yet. To gain insight into which neuronal populations and specific circuits may be influenced by Reelin in the adult spinal cord, we have conducted light and confocal microscopy study analysis of Reelin-immunoreactive cell types in the adult spinal cord. Here, we describe and compare Reelin immunoreactive cell type and distribution in the spinal cord of adult non-human primate (macaque monkeys, *Macaca mulatta*), carnivore (ferret, *Mustela putorius*) and rodent (rat, *Rattus norvegicus*). Our results show that in all three species studied, Reelin-immunoreactive neurons are present in the intermediate gray matter, ventricular zone and superficial dorsal horn and intermedio-lateral nucleus, while positive cells in the Clarke nucleus are only found in rats and primates. In addition, Reelin intermediolateral neurons colocalize with choline acetyltransferase (ChAT) only in macaque whilst motor neurons also colocalize Reelin and ChAT in macaque, ferret and rat spinal cord. The different expression patterns might reflect a differential role for Reelin in the pathways involved in the coordination of locomotor activity in the fore- and hind limbs.

## Introduction

Reelin is a large extracellular matrix (ECM) molecule that is crucial for the neuronal migrations involving the laminar organization of different brain regions and sympathetic preganglionic neurons during development (Tissir and Goffinet, 2003; Jossin, 2004; Niu et al., 2004; Yip et al., 2007; Krüger et al., 2010). In the adult forebrain, Reelin signaling has been associated with dendritic growth and postsynaptic events during long-term potentiation (Goffinet, 1984; Haas et al., 2002; Hack et al., 2002; Ohshima et al., 2002; Weeber et al., 2002; Niu et al., 2004; Cariboni et al., 2005). Other studies have suggested an involvement of Reelin signaling in pain processing in the spinal cord (Villeda et al., 2006; Akopians et al., 2008; Wang et al., 2012). However, the distribution of cells expressing Reelin in the adult spinal cord has been examined fragmentarily, and only in rodents (Phelps et al., 1991; Kubasak et al., 2004; Villeda et al., 2006). Here we describe and compare Reelin immunoreactivity in the spinal cord of adult non-human primate *(Macacca nemestrina)*, carnivore *(Mustela putorius)* and rodent *(Rattus norvegicus)*. Our results suggest that Reelin is involved and conserved in adult nociceptive pathways across phylogenetically separate mammalian species. In addition, some neurons in proprioceptive and motor spinal pathways also express detectable levels of Reelin in some species, but not in others.

## Materials and Methods

### Animals and Anesthetic Procedures

Spinal cord tissue from three adult macaques (*Macaca mulatta*), three adult pigmented ferrets (*Mustela putorius furo*) and 12 Sprague–Dawley adult rats were used for the present study. Procedures involving live animals were carried out in accordance with the European Community’s Council Directive 86/609/EEC, and NIH guidelines, and approved by our University’s Bioethics Committee. Animals were overdosed with sodium pentobarbital (80 mg/kg, intraperitoneal), and subsequently perfused intracardially with saline, followed by 4% paraformaldehyde in 0.1 M phosphate buffer (PB; pH 7.4).

### Perfusion and Histology

After perfusion, spinal cords were removed and tissue was cryoprotected by immersing in 30% sucrose in PB for 24–48 h at 4°C. No post-fixation was made. Blocks at three levels of the spinal cord were obtained (cervical, thoracic and lumbar) and parallel series of 60 μm thick were cut on a freezing microtome. Rexed’s (1954) and Paxinos and Watson’s (1998) criteria were followed for the delineation of the laminas. Immunolabeled slices were compared with adjacent slices stained for Nissl substance with Cresyl Violet.

### Immunohistochemistry

Two antibodies were used for the Reelin immunohistochemistry, G142 (Calbiochem, 1:400) and CR50 (a gift of Dr. M. Ogawa, RIKEN, Japan), both raised in mouse. A biotinylated rabbit anti-mouse IgG (1:200, Chemicon) was used as the secondary antibody. The sections were subsequently incubated in avidin-biotinylated horseradish peroxidase complex (ABC, Vector Laboratories, Burlingame, CA, USA) in 0.1 M PBS for 1 h, and developed with 0.01% H_2_O_2_ + 0.04% 3,3′-diaminobenzidine tetrahydrochloride (DAB). The sections used for immunofluorescence were incubated with G142 (Calbiochem, 1:400) and goat anti choline acetyltransferase (ChAT, Millipore; 1:200) and fluorescent secondary antibodies: donkey anti-goat secondary antibody (Alexa Fluor 488, Molecular Probes, 1:1,000) and donkey anti-mouse (Alexa Fluor 647, Molecular Probes, 1:1,000). The omission of primary antibodies was included as a control for immunolabeling specificity. The sections were mounted onto gelatin-coated glass slides and air-dried. Finally, ABC developed slices were dehydrated in graded alcohols, cleared in xylene, and cover-slipped with DePeX. Immunofluorescent slides were cover-slipped with Mowiol.

### Imaging and Data Analysis

DAB-stained sections were examined and photographed under bright and dark-field illumination using a Nikon 600 Eclipse microscope under 4–40× Wide-Diameter Plan-Apochomat Nikon objectives. Images were acquired with a Nikon DXM 1200 Brightness and gamma adjustment of the images was made using CANVAS X software.

To analyze double-labeled sections, pictures were carried out on optical slices made with a Leica TCS-SPII spectral confocal microscope by sequentially applying different laser lines (Argon-Ion; Helio-Neon) to ensure complete channel separation. This analysis was conducted in the three different species in all spinal cord levels: cervical, thoracic and lumbar. We examined single 3 μm-thick optical slice per ROI where we determined co-localization by plotting the position of the labeled cell profiles on the single-channel confocal images and then overlaying the plots. Parallel tissue samples developed without primary antibodies showed no immunolabeling. Rexed’s (1954) and Paxinos and Watson’s (1998) criteria were used for the delineation of the laminas and the nuclei.

## Results

### Reelin Protein Pattern in the Dorsal Horn

**Figure 1:**
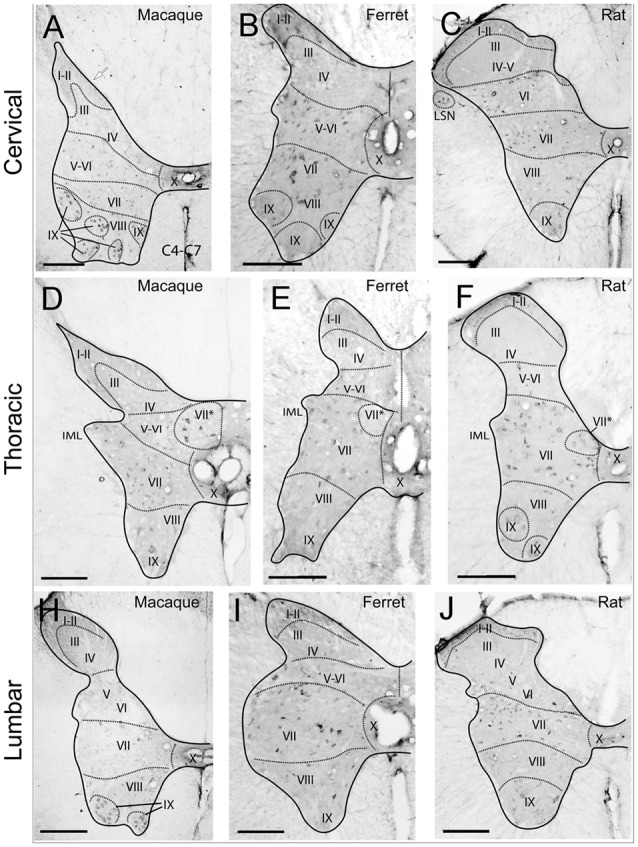
Immunoreactivity to Reelin of cervical, thoracic and lumbar sections in macaque, ferret, and rat. Reelin immunoreactivity pattern in the cervical **(A–C)**, thoracic **(D–F)** and lumbar **(H–J)** sections from macaque **(A,D,H)**, ferret **(B,E,I)** and rat **(C,F,J)** spinal cords. (I–X) refers to the different Rexed’s laminae (I–X) throughout the different sections. VII*: Clarke’s column. *Calibration bar*: **(A,H)** 500 μm; **(B–G,I,J)** 250 μm.

**Figure 2:**
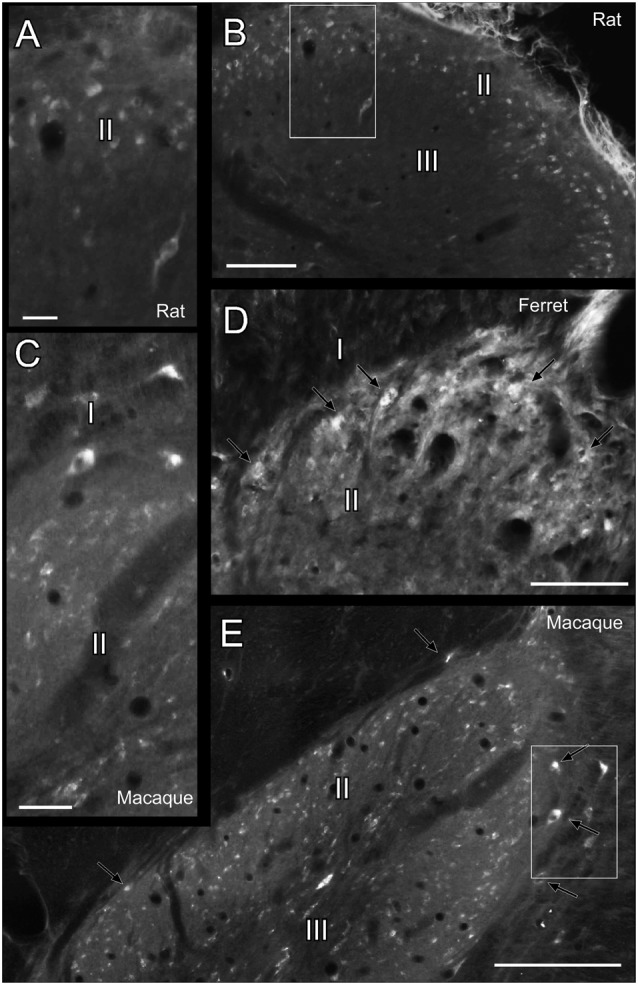
Reelin labeling pattern in the dorsal horn of the spinal cord. **(A)** High magnification of box in **(B)**. **(B)** Dorsal horn of the spinal cord in a rat cervical section. **(C)** Detail of the box in **(E)**. **(D)** Dorsal horn of the spinal cord in a ferret cervical section. **(E)** Dorsal horn of the spinal cord in a macaque cervical section. I, II, III refers to Rexed’s laminae I, II and III, respectively. Arrows point to immunoreactive cells resembling Waldeyer cells.* Calibration bar*: **(B,D,E)** 100 μm; **(A,C)** 25 μm.

The studied species hold Reelin immunoreactive neurons in the superficial layers of the dorsal horn and are scattered throughout deeper dorsal laminas throughout all spinal levels (Figures 1A–H). Reelin immunoreactive cells are small and round in laminas I–II in the three species (Figures 2A–E) and, in non-human primates and carnivores, some of the immunoreactive cells are bigger and resemble Waldeyer cells, a specific population of lamina I projection neurons conveying noxious information to the brain (Puskár et al., 2001; Figures 2C,D). Moreover, we also observe strong immunoreactive neuropil in the superficial layers of the dorsal horn, which is most evident in the ferret (Figures 2A,C,D). Reelin immunoreactive cells are also found in the lateral spinal nucleus (LSN) in rats (Figure 1C).

### Pattern of Reelin Protein in the Lateral Horn

**Figure 3:**
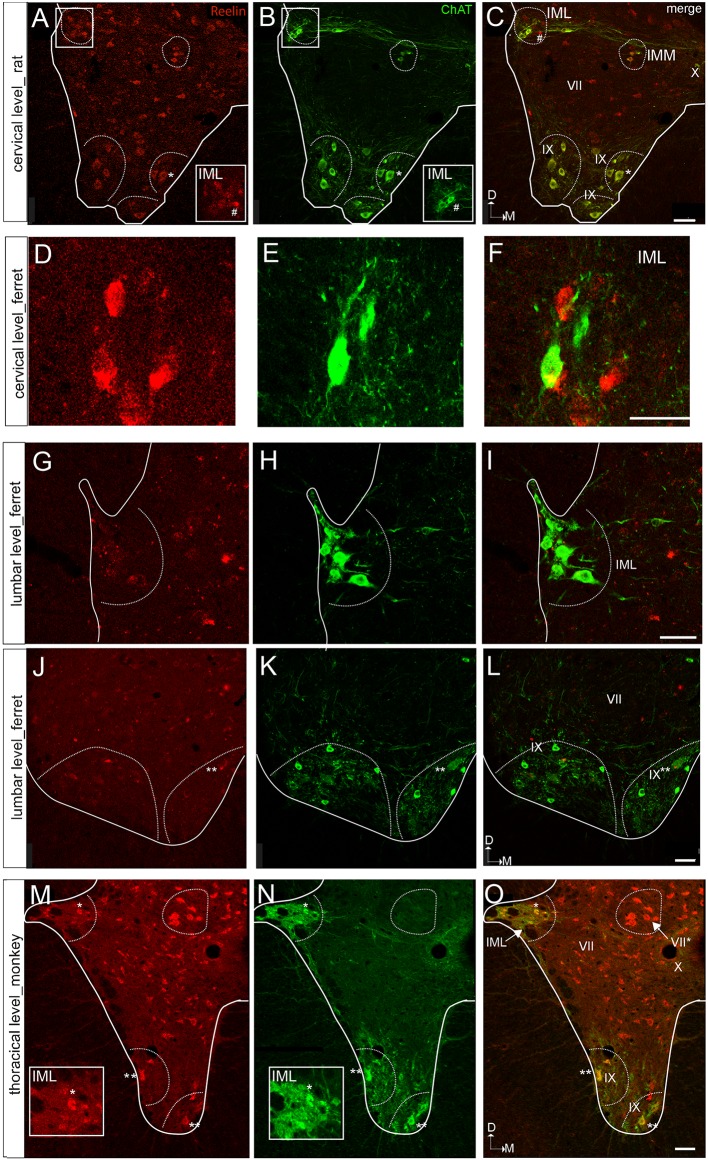
Reelin and choline acetyltransferase (ChAT) immunostaining of the ventral horn in the rat, ferret, and macaque. **(A–C)** Spinal cord section of a rat at a cervical level showing ChAT colocalization with Reelin immunoreactive cells in the IMM and lamina IX, the latest corresponding to motor neurons. In contrast, Reelin immunoreactive cells do not coexpress ChAT in the IML. **(D–F)** Spinal cord section of a ferret at a cervical level showing that Reelin immunoreactive cells are not positive for ChAt in the IML. **(G–I)** Spinal cord section of a ferret IML at the lumbar level. **(J–L)** Spinal cord section of a ferret at a lumbar level showing a faint immunoreactivity for Reelin in the motor neurons of lamina IX. **(M–O)** Spinal cord section of a macaque at a thoracic level showing a strong immunoreactivity for Reelin in the motor neurons of lamina IX and in the IML. VII, IX and X correspond to laminae VII, IX and X, respectively. IML, intermediolateral nucleus; IMM, intermedio-medial nucleus. *Calibration bar*: **(A–C)** 50 μm; **(D–F)** 40 μm; **(G–I)** 50 μm; **(J–L)** 75 μm; **(M–O)** 50 μm.

**Figure 4:**
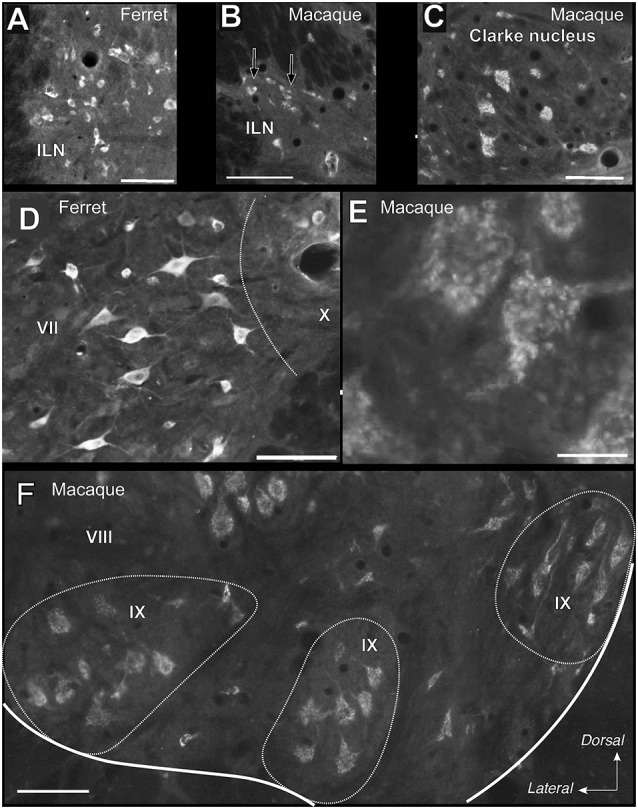
High magnifications of the immunoreactive cells in the ventral and lateral horn of the spinal cord in macaque and ferret. **(A)** Immunoreactive cells in the intermediate lateral nucleus (ILN) of a ferret thoracic section. **(B)** Small labeled neurons in the ILN of a lateral horn in a macaque thoracic section. Arrows indicate immunoreactive neurons. **(C)** Labeled neurons in the Clarke nucleus in lamina VI of a macaque thoracic section. **(D)** Reelin-positive cells located in the intermediate zone (intermedio-medial cell column) in a ferret lumbar section. **(E)** The speckled appearance of Reelin positive cells located in the ventral horn of a macaque lumbar section. **(F)** Distribution of Reelin positive cells located in the lamina IX in a macaque cervical section. *Calibration bar*: **(A–D,F)** 100 μm; **(E)** 25 μm.

As for the dorsal horn, immunoreactive cells are found throughout all levels in the lateral horn. Specifically, Reelin immunoreactivity is present in the preganglionic neurons of the intermediate lateral nucleus (ILN) in a non-human primate, ferret and rat and immunolocalize with ChAT in the non-human primates (Figures 3A–L, 4D–F). As for the ferret, Reelin immunoreactivity is low or absent in the same nucleus when lumbar levels are reached (Figures 1B,E,I, 3D–I, 4A,B).

On the other hand, Reelin-positive medium-sized cells are present in lamina VII in all studied species although, the ferret again the exception, large Reelin-positive cells are present in the origin of the dorsal spinocerebellar tract, the *Clarke* nucleus (VII*; Figures 1D,F, 3D–F, 4C).

### Pattern of Reelin Protein in the Ventral Horn

Large Reelin-immunoreactive cells are found in laminas VIII and IX in a non-human primate, ferret and rat (Figures 1A–J, 3A–L, 4D–F). Immunoreactive speckled neurons in lamina IX can be found in all studied species although the expression levels of Reelin varies amongst species. The highest expression is found in non-human primate at all levels (Figures 1A–J, 3A–C,G–L, 4E,F). In addition, these Reelin immunoreactive cells colocalize with ChAT in lamina IX in all three studied species (Figures 3A–C,G–O).

## Discussion

We examined the Reelin immunolabeling pattern in the adult spinal cord of three widely used laboratory model species of carnivore, rodent and non-human primate. Our observations reveal a basic similar pattern of Reelin immunostaining in the three species. Numerous Reelin-positive neurons are present in the intermediate gray matter and the superficial dorsal horn while the dorsal and ventral commissure close to the floor plate is devoid of Reelin-positive cells. In addition, Clarke nucleus in rats and primates contains Reelin-reactive cells and preganglionic cells positive for Reelin are found in the ILN of all studied species. Finally, some motor neurons in rats and primates show a speckled intracellular staining pattern previously observed for the motor neurons in the medulla of non-human primate (Martínez-Cerdeño et al., 2002).

### Specificity of the Reelin Immunolabeling

Here we confirm the reported distribution of Reelin in the spinal cord from previous studies focusing on the function and expression of Reelin during the development of the spinal cord in rodents (Yip et al., 2000, 2004, 2007; Phelps et al., 2002; Kubasak et al., 2004; Villeda et al., 2006) and extend it to the adulthood in rodents, carnivores, and macaques.

The specificity of the monoclonal antibodies used in the present study has been extensively characterized and reported in previous studies by our group addressing the distribution of Reelin in the adult brain of Sprague–Dawley rats, ferrets (*Mustela Pistorius*) and non-human primates (*Macaca mulatta*; de Bergeyck et al., 1997; Martínez-Cerdeño and Clascá, 2002; Martínez-Cerdeño et al., 2002, 2003; Ramos-Moreno et al., 2006), the same animal models that are used in the present work for characterizing the distribution of Reelin in the spinal cord. These monoclonal antibodies are two IgGs that are directed to adjacent but non-overlapping amino acid critical sequences for protein function in the Reelin F-spondin-like region (Ogawa et al., 1995; Del Río et al., 1997; Nakajima et al., 1997; de Bergeyck et al., 1998; Borrell et al., 1999; Utsunomiya-Tate et al., 2000; Ichihara et al., 2001; Quattrocchi et al., 2002) and possess high affinity for the full-length protein and processed forms in rodents and primates (de Bergeyck et al., 1998; Impagnatiello et al., 1998; Hong et al., 2000; Lacor et al., 2000). Therefore, it is unlikely that the two monoclonal IgGs would react with a protein other than Reelin. Finally, the omission of the primary antibody yielded no labeling.

The speckled pattern of Reelin immunoreactivity observed in motor neurons in our study resembles that of the Golgi apparatus, previously described as an elongated and irregular pattern in layer V cortical neurons in rat and primates and confirmed by electron microscopy studies (Martínez-Cerdeño et al., 2002; Ramos-Moreno et al., 2006). Previous studies have suggested that Reelin protein may be found in: (1) the endoplasmic reticulum and Golgi complex of synthesizing neurons (Pappas et al., 2001; Martínez-Cerdeño et al., 2002); (2) in membrane-bound vesicles within axons as it is transported for secretion (Derer et al., 2001; Martínez-Cerdeño et al., 2002, 2003; Pappas et al., 2003); (3) in the ECM and attached to cell membranes of synaptic neuropils after secretion (D’Arcangelo et al., 1997; Pappas et al., 2001; Dong et al., 2003); (4) re-internalized after binding to receptors in target cells (D’Arcangelo et al., 1999; Morimura et al., 2005); and (5) the intercellular space (Sáez-Valero et al., 2003; Ignatova et al., 2004). However, we cannot exclude the possibility of other options for the subcellular localization of Reelin in the spinal cord neurons (specifically, that of Reelin re-internalization, see below).

### Reelin Presence in the Spinal Cord in Different Species

Present results confirm previous observations in pre- and postnatal rodent’s spinal cord (Phelps et al., 2002; Kubasak et al., 2004; Villeda et al., 2006) and extend the available data to adult non-human primates, carnivores and rodents. For example, we observe the high concentration of diffuse extracellular Reelin reactivity and Reelin-positive cells in laminae I, II in all studied species where nociceptive information is relayed (Chaouch and Besson, 1986; Aziz et al., 2009). The first evidence for a possible role of Reelin in nociception comes from the *reeler* mouse where the anatomical abnormalities were suggested to have functional consequences and be responsible for the significant reduction in mechanical sensitivity and the pronounced thermal hyperalgesia described in the mutant (Villeda et al., 2006). Reelin pathways involved in nociception in adulthood concluded that the Reelin-Dab1 pathway contributes to acute and persistent pain (Akopians et al., 2008; Wang et al., 2012).

Our work also confirms the presence of Reelin in the adult ILN in rodents and extends its presence to the ILN of adult carnivores and non-human primates. However, Reelin colocalized with ChAT only in macaques, thus it can be concluded that Reelin is present in preganglionic ILN cells only in non-human primates. The exclusion of preganglionic cells as a Reelin source has been previously reported during the spinal cord development in rodents, where the preganglionic cells have been reported to not express the Reelin mRNA but to express the VLDLR and APOE Reelin receptors (Yip et al., 2000, 2004; Phelps et al., 2002; Lee and Song, 2013). In the adult, preganglionic cells are involved in the regulation of the endocrine system and smooth muscles. The different expression of Reelin between species is likely to reflect the species differences between primates and other mammals regarding the different regulation of the physiology between species, which has been reported previously (Phillips et al., 2014). A species difference is also supported by the augmented gene expression of genes in the primate’s central nervous system (Naumova et al., 2013) which can include Reelin (discussed in Martínez-Cerdeño et al., 2002). That Reelin can be part of the modulation of smooth musculature in primates but not in other species should be tested. The absence of Reelin in knock out mouse models has been reported to be responsible for the impairment of vessel morphogenesis and function (Lutter et al., 2012).

Likewise, present results confirm the presence of Reelin positive cells in Clarke’s column in rodents and extend it to primates, but not to carnivores. Clarke’s column is involved in proprioception and is involved in Friedrich’s ataxia (Haines, 2008). Patients suffering from this disease develop ataxia, dysarthria, muscle weakness or paralysis, and skeletal defects, thus resembling features described in the *reeler* mutant (D’Arcangelo et al., 1995). Reelin immunoreactive interneurons in the lamina VII intermediate zone may play a role in limb coordination (gait control), as this is the typical function described to be for this lamina (Blumenfeld, 2010). Nevertheless, it is noteworthy that Reelin is only present in Clarke’s nucleus in primates and rodents. The latest could again reflect a different physiology between species. In primates and rodents but not in carnivores, spinocerebellar afferents are found in Clarke’s nucleus in the sacral and coccygeal segments, which receive a powerful input from passive movements of the tail (Milne et al., 1982; Kayalioglu, 2009).

Finally, motor neurons in the non-human primate ventral horn clearly contain Reelin, while the staining is faint in adult rats and carnivores. Previous mRNA studies in rodent spinal cord during postnatal stages and development showed that motor neurons do not express Reelin (Phelps et al., 2002; Kubasak et al., 2004). A possible explanation for Reelin expression in the adult motor neurons could be the reinternalization of the Reelin protein and anterograde axoplasmic transport. The hypothesis that Reelin can be re-internalized by neuronal cells from other sources has been previously suggested upon observations of long-distance effects of Reelin (Martínez-Cerdeño et al., 2003; Ramos-Moreno et al., 2006; Jossin et al., 2007). On the other hand, the above-mentioned mRNA expression studies were performed in developmental and postnatal stages and we cannot exclude the possibility that Reelin mRNA starts to be expressed in the adult motor neurons. Moreover, poor sensitivity of the probe used for the detection of mRNA or low performance of the probe on thick tissue sections cannot be ruled out (Femino et al., 1998; Speel et al., 1999; Ramos-Moreno et al., 2006). Only negative results using PCR amplification can effectively rule out the presence of low, but biologically significant, numbers of mRNA transcripts and, because of post-transcriptional regulatory mechanisms, low mRNA levels do not directly imply low protein levels (Gygi et al., 1999; Tian et al., 2004). Of note is that protein and mRNA abundances are determined by the relationships between the rates of the processes producing and degrading the participating molecules. In mammals, mRNAs are produced at a much lower rate than proteins are (Vogel and Marcotte, 2012). Structural proteins, such as ECM proteins, are in addition, longer-lived thus requiring less mRNA (Vogel and Marcotte, 2012). This supports the notion that a low abundance of Reelin mRNA should not exclude a possible high content of Reelin protein. A study of Reelin mRNA expression in the adult would be of interest.

One way or another, Reelin seems to be present in pathways involved in synchronizing lumbar and cervical pattern generators and hence the coordination of locomotor activity in the fore- and hind limbs (Brockett et al., 2013). The latest support the motor symptoms observed in animals lacking the Reelin protein (D’Arcangelo et al., 1995; de Bergeyck et al., 1997). Moreover, our data support the notion of an increased presence of Reelin in the central nervous system of primates, this being involved in motor pathways that, according to our present data, can be playing a role for controlling both smooth and striated musculature as well as being involved in the coordination of movements. Reelin could thus lead to novel strategies for treating ataxias.

## Data Availability Statement

The datasets generated for this study are available on request to the corresponding author.

## Ethics Statement

The animal study was reviewed and approved by the Autónoma de Madrid University Bioethics committee and the competent Regional Government agency (PROEX175/16, PROEX189/16). Procedures were carried out in accordance with the European Community’s Council Directive 86/609/EEC, and NIH guidelines.

## Author Contributions

AK: investigation, analysis, resources and funding acquisition, review and editing of manuscript. MC: investigation, review, and editing of manuscript. FC: conceptualization, resources, funding acquisition, review and editing of manuscript. TR-M: conceptualization, investigation, methodology, analysis, supervision, funding acquisition, and writing of the manuscript.

## Conflict of Interest

The authors declare that the research was conducted in the absence of any commercial or financial relationships that could be construed as a potential conflict of interest.
